# Estimation of sarcopenia prevalence in individuals at different ages from Zheijang province in China

**DOI:** 10.18632/aging.202567

**Published:** 2021-02-18

**Authors:** Jie Huang, Fan He, Xue Gu, Shoushun Chen, Zhendong Tong, Suya Zhong

**Affiliations:** 1 The Second Affiliated Hospital of Zhejiang Chinese Medical University, Hangzhou, Zhejiang, China; 2 Zhejiang Provincial Center for Disease Control and Prevention, Hangzhou, Zhejiang, China; 3 Putuo Mountain Community Center for Health and Service, Zhoushan, Zhejiang, China; 4 Zhoushan Center for Disease Control and Prevention, Zhoushan, Zhejiang, China

**Keywords:** sarcopenia, skeletal muscle index, prevalence, cut-off point, body composition

## Abstract

In this study, we analyzed sarcopenia prevalence and the cut-off points for skeletal muscle mass index (SMI), gait speed, and handgrip strength in young (18-39 years), middle-aged (40-59 years), and elderly (>60 years) individuals (n=1685) from Zhejiang Province in China. The prevalence of sarcopenia among individuals above 65 years was 2.21%, 4.87%, 5.31%, 14.16%, and 16.37% according to five diagnostic criteria (AWGS2019, AWGS2014, EWGSOP1, EWGSOP2, and local standard). The mean SMI (Kg/m^2^) was 7.961±0.7966, 7.801±0.7276, and 7.544±0.7493, respectively, in young, middle-aged, and elderly males. The mean SMI in young, middle-aged, and elderly females was 6.1570±0.5658, 6.604±0.5658, and 6.248±0.7483, respectively. SMI correlated negatively with age (r=-0.2344, P<0.001), but was not associated with age in females (r=0.0573, P=0.1463). The cut-off point of SMI for sarcopenia was ≤6.3678 kg/m^2^ in males and ≤5.0254 kg/m^2^ in females. These findings shows that the prevalence of sarcopenia increased gradually with age and varied significantly based on the diagnostic criteria used for this analysis. The mean SMI of young women was lower than in middle-aged women, making them an unsuitable reference population for determining cut-off values for sarcopenia diagnosis.

## INTRODUCTION

Sarcopenia is an age-related syndrome that is characterized by progressive loss of muscle mass, strength and function that reduces quality of life and is accompanied by physical disability [1]. It was officially recognized in 2016 as a specific disease and assigned the diagnosis and staging code, ICD-10-CM (M62.84) [2, 3] The prevalence of sarcopenia is 5-13% among individuals aged 60-70 years [4, 5] and 11-50% among individuals aged above 80 years [6, 7].

Muscle mass varies among people of different racial origin [8], and is influenced by genes [9], hormones [10], and lifestyle [11]. Sarcopenia is defined as by reduction in skeletal muscle mass that is two standard deviations below the average muscle mass of young adults as the reference population [1, 7, 12–14]. As a result, diagnosis and prevalence results of sarcopenia vary significantly between populations because the cut-off values are different. Despite similar socioeconomic backgrounds, there is great variation in body composition as well as muscle mass, strength and performance between individuals of different races [15]. Hence, the cut-off values for the diagnosis of sarcopenia based on muscle mass, strength, and function need to be determined based on ethnicity [16]. Currently, the cut-off points for the diagnosis of sarcopenia are arbitrary due to differences in measurement techniques and reference populations. The European Working Group on Sarcopenia in older people (EWGSOP2) [17] and the Asian Working Group on Sarcopenia (AWGS) in 2014 [12] and 2019 [18] proposed key criteria for the diagnosis of sarcopenia in order to compare results of studies involving individuals from different ethnic backgrounds.

EWGSOP2 used muscle strength as the main parameter for diagnosing sarcopenia, but, evaluation of muscle loss varies considerably based on the instruments and methods that are used [15] Currently, the cut-off points for the diagnostic parameters of sarcopenia are arbitrary because of differences in measurement techniques and reference populations. In China, the parameters proposed by AWGS in 2014 have been used for sarcopenia studies [19, 20]. The prevalence of sarcopenia in older adults is 17% in China compared to 6% in Hong Kong and Taiwan [21].

The cut-off points for determining loss of muscle mass varies significantly in studies conducted in different Chinese populations [5, 22, 23]. Moreover, most of the data regarding sarcopenia is obtained from the elderly individuals and groups, and very few studies have monitored the prevalence of sarcopenia in younger Chinese populations. In particular, the data for muscle mass is not available for young adults. Sarcopenia is common among older adults but can also occur in younger and middle aged individuals. Hence, development of validated cut-off points based on normative data and their predictive value is of high priority [17]. This requires analysis of the relevant diagnostic parameters of sarcopenia in different age groups.

In this study, we evaluated the cut-off point for the skeletal muscle index to diagnose sarcopenia in 1685 Chinese individuals belonging to different age groups from the Zhejiang Province. We also compared the differences in sarcopenia prevalence among males and females belonging to different age groups based on five different diagnostic criteria.

## RESULTS

The prevalence of sarcopenia based on the five diagnostic criteria ranged from 2.61%-9.72% for individuals aged above 60 years, 4.87%-16.37% for individuals aged above 65 years, and 18.52%-55.56% for individuals aged above 80 years old. Overall, sarcopenia prevalence increased significantly with age (P<0.001, Table 1). Moreover, the prevalence of sarcopenia among females in different age groups evaluated according to the EWGSOP2 parameters was statistically similar (P=0.345, Table 1).

**Table 1:** Prevalence of sarcopenia in males and females belonging to different age groups according to the 5 diagnostic criteria.

**Gender**	**Diagnostic criteria**	**Age group (% sarcopenia positive)**					**Chi-square**	**P value**
		**18-39y**	**40-59y**	**≥60y**	**≥65y**	**≥80y**		
Male	AWGS2019	0.32 (1/311)	1.85 (10/541)	6.67 (16/240)	12.9 16/124)	53.85 (7/13)	71.134	<0.001
	AWGS2014	0.32 (1/311)	0.37 (2/541)	5.83 (14/240)	10.48 (13/124)	38.46 (5/13)	74.491	<0.001
	EWGSOP 1	0.32 (1/311)	0.37 (2/541)	2.92 (7/240)	5.65 (7/124)	23.08 (3/13)	74.491	<0.001
	EWGSOP2	0.32 (1/311)	0 (0/541)	1.67 (4/240)	3.23 (4/124)	15.38 (2/13)	25.436	<0.001
	Local standard	0.32 (1/311)	0 (0/541)	2.5 (6/240)	4.84 (6/124)	15.38 (2/13)	32.869	<0.001
Female	AWGS2019	0.43 (1/230)	1.1 (2/181)	13.74 (25/182)	20.59 (21/102)	57.14 (8/14)	77.934	<0.001
	AWGS2014	0.43 (1/230)	1.1(2/181)	3.85 (7/182)	18.63 (9/102)	57.14 (8/14)	91.299	<0.001
	EWGSOP 1	0 (0/230)	0 (0/181)	2.75 (5/182)	4.9 (5/102)	21.43 (3/14)	91.299	<0.001
	EWGSOP2	0 (0/230)	0 (0/181)	0.55 (1/182)	0.98 (1/102)	0 (0/14)	4.81	0.345
	Local standard	0 (0/230)	0 (0/181)	2.75 (5/182)	4.9 (5/102)	21.43 (3/14)	26.888	<0.001
Total	AWGS2019	0.37(2/541)	1.66 (12/722)	9.72 (41/422)	16.37 (37/226)	55.56 (15/27)	150.268	<0.001
	AWGS2014	0.37(2/541)	0.55 (4/722)	4.98 (21/422)	14.16 (32/226)	48.15 13/27)	171.028	<0.001
	EWGSOP 1	0.18 (1/541)	0.28 (2/722)	2.84 (12/422)	5.31 (12/226)	22.22 (6/27)	171.028	<0.001
	EWGSOP2	0.18 (1/541)	0(0/722)	1.18 (5/422)	2.21 (5/226)	7.14 (2/27)	24.568	<0.001
	Local standard	0.18 (1/541)	0(0/722)	2.61 (11/422)	4.87 (11/226)	18.52 (5/27)	60.644	<0.001

As shown in Table 1, the prevalence of sarcopenia as calculated by the five different diagnostic criteria varied widely. In both males and females, the highest percentage of sarcopenia prevalence was according to AWGS 2019 and the lowest percentage was according to EWGSOP2. The percentages of sarcopenia prevalence according to AWGS 2014, EWGSOP1, and local standard were in between the values obtained for AWGS 2019 and EWGSOP2. This demonstrated that the clinical evaluation of sarcopenia prevalence varied significantly based on the diagnostic criteria used for analysis.

**Figure 1:**
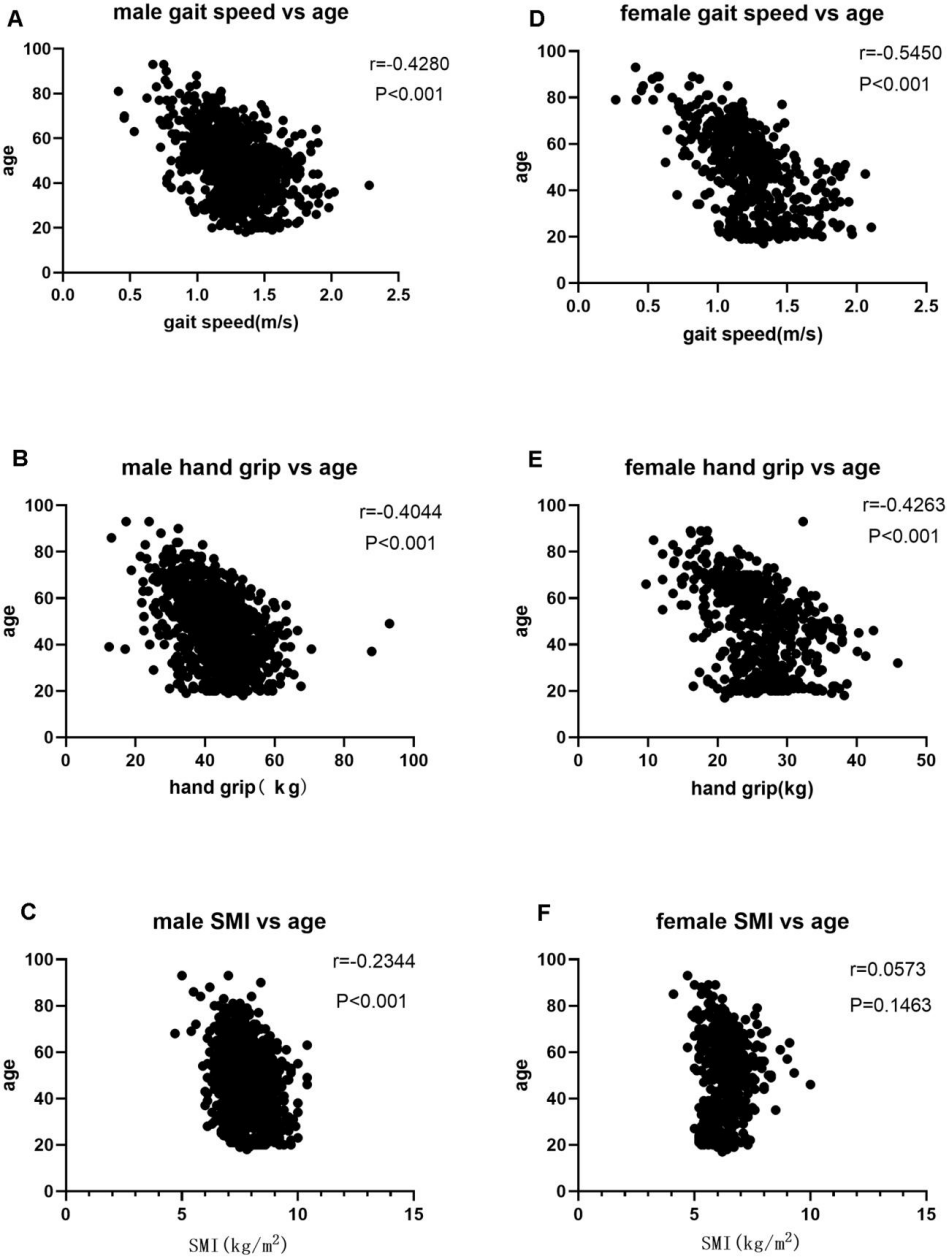
(**A**) Correlation analysis of gait speed and age in males; (**B**) Correlation analysis of hand grip and age n males; (**C**) Correlation analysis of SMI and age in males; (**D**) Correlation analysis of gait speed and age in females; (**E**) Correlation analysis of hand grip and age in females; (**F**) Correlation analysis of SMI and age in females.

We then performed Pearson correlation analysis according to gender stratification to determine the relationship between age and sarcopenia parameters such as SMI, gait speed, and hand grip. In males, we observed a negative correlation between age and SMI (r=-0.2344), hand grip (r=-0.4044), and gait speed (r=-0.4280) (P<0.001; Table 2, Figure 1A–1C). In females, we did not observe any association between SMI and age (r=0.0573, P=0.1463, Table 2, Figure 1D–1F), but observed a negative correlation between age and hand grip (r=-0.4263) and gait speed (r=-0.5450) (P<0.001; Table 2, Figure 1D, 1E).

**Table 2:** Pearson correlation analysis between diagnostic indicators of sarcopenia and age in males and females.

**Gender**	**Parameters**	**r**	**P**
Male	gait speed	-0.4280	<0.001
	Hand grip	-0.4044	<0.001
	SMI	-0.2344	<0.001
Female	gait speed	-0.5450	<0.001
	Hand grip	-0.4263	<0.001
	SMI	0.05973	0.146

**Figure 2:**
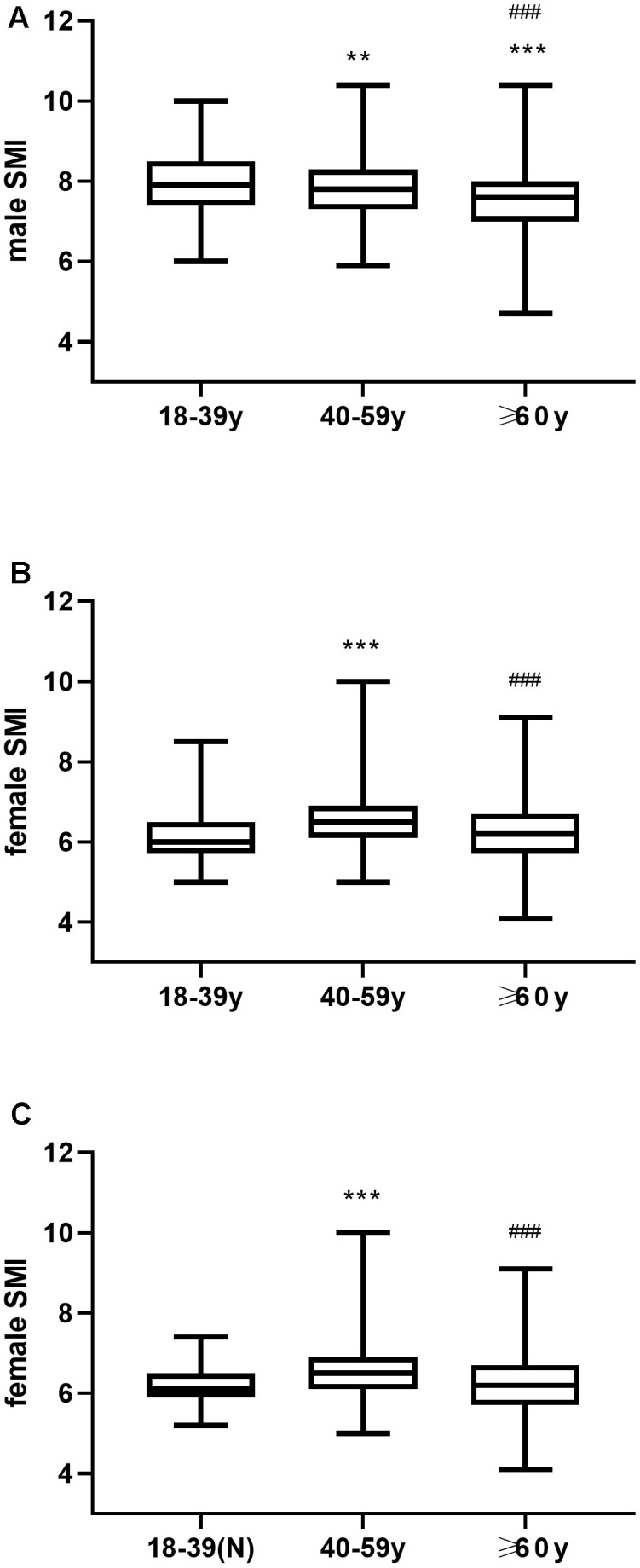
(**A**) Mean SMI in young, middle-aged and elderly men. (**B**) Mean SMI in young, middle-aged and elderly women. (**C**) Mean SMI in young women with normal BMI (18.5-23.9) as well as middle-aged and elderly women. ** P ≤0.01 vs 18-39y, *** P <0.001 vs 18-39y, ^###^ P <0.001 vs 40-59y.

As shown in Figure 2, the mean SMI values for men and women aged 18-39 years were 7.961 ± 0.7966 kg/m^2^ and 6.157 ± 0.5658 kg/m^2^, respectively. Therefore, the cut-off points for the abnormal SMI (two standard deviations below the mean SMI) in males and females aged 18-39 years were ≤ 6.3678 kg/m^2^ and ≤ 5.0254 kg/m^2^, respectively. These cut-off points were significantly lower than the recommended AWGS values for SMI, namely, ≤ 7.0 kg/m^2^ for males and ≤ 5.7 kg/m^2^ for females.

In males, the SMI values gradually decreased with age, and the pair-wise comparisons between young, middle-aged, and elderly age groups were statistically significant (P<0.001, Figure 2A). Moreover, young women and young women with normal BMI showed significantly lower SMIs than the middle-aged women (P < 0.001, Figure 2B, 2C), and lower but statistically similar SMI values than the elderly women (P=0.34 Figure 2, P>0.05, Figure 2B). Hence, young women were not suitable as a reference population for determining the cut-off point to diagnose sarcopenia.

As shown in Table 3, the mean SMI values for women aged 60-69 years were 6.386 ± 0.7492 kg/m^2^. The females aged 60-69 years showed significantly higher SMI values than the females aged 18-29 years (P=0.0031, Table 3), and SMI values were statistically similar, compared with the females aged 30-39, 40-49, 50-59, 18-39, 18-59 years (P>0.9999, P=0.9286, P>0.9999 P= 0.0689, P>0.9999, Table 3). The females aged>60 years showed significantly similar SMI values than the females aged 18-29, 18-39 years (P=0.4326, P> 0.9999, Table 3). The females aged 18-29 years showed significantly similar SMI values than the females aged 18-39 years (P=0.1911, Table 3).

**Table 3:** Comparison of SMI values among women in different age groups.

**Age**	**mean1**	**mean2**	**Summary**	**P value**
18-29y vs. 60-69y	6.074	6.386	**	0.0031
30-39y vs. 60-69y	6.325	6.386	ns	>0.9999
40-49y vs. 60-69y	6.604	6.386	ns	0.9286
50-59y vs. 60-69y	6.604	6.386	ns	>0.9999
18-39y vs. 60-69y	6.157	6.386	ns	0.0689
18-59y vs. 60-69y	6.354	6.386	ns	>0.9999
18-29y vs. ≥60y	6.074	6.248	ns	0.4326
18-39y vs. ≥60y	6.157	6.248	ns	>0.9999
18-29y vs. 30-39y	6.074	6.325	ns	0.1911

**Figure 3:**
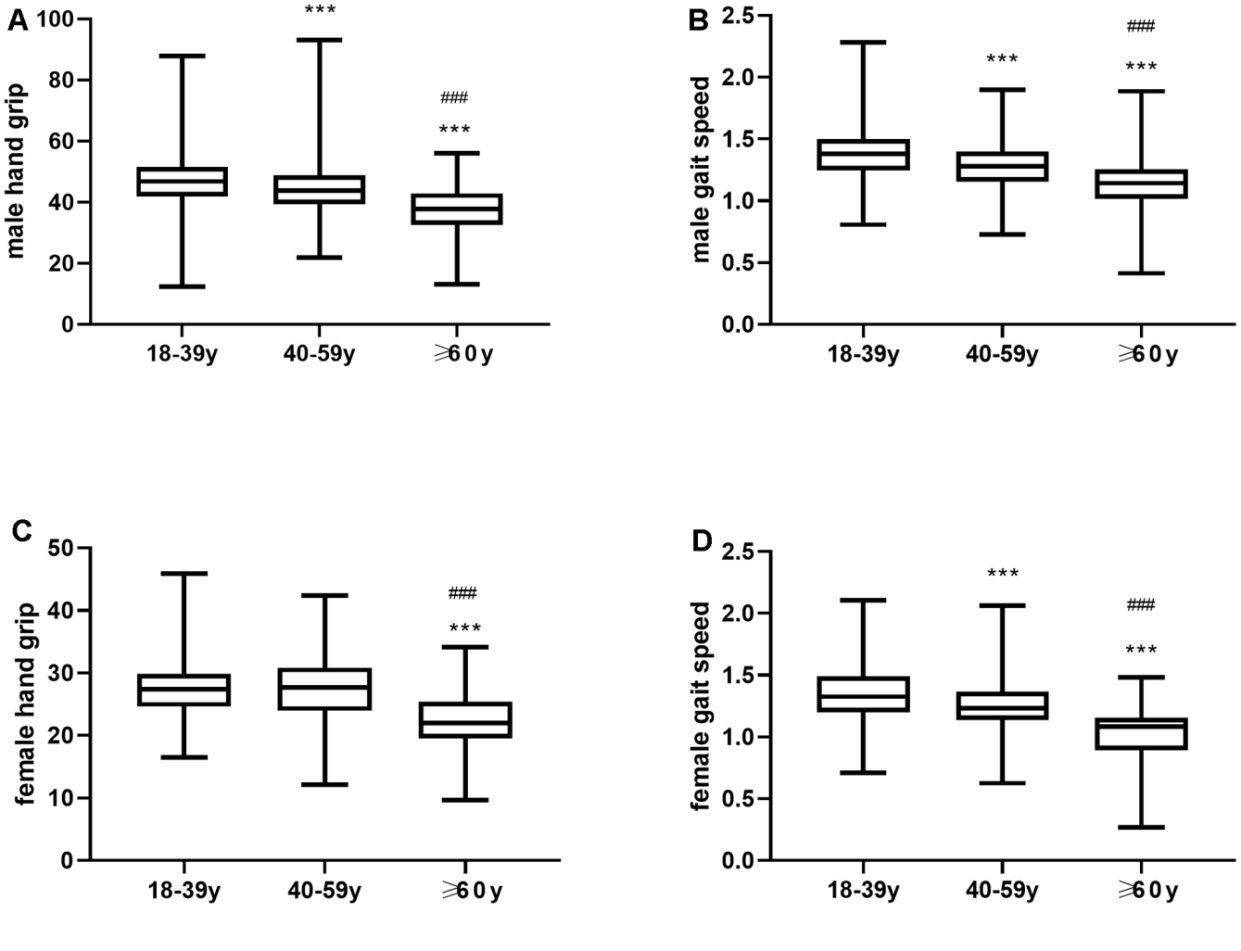
(**A**) Mean hand grip values and (**B**) Mean gait speed in young, middle-aged and elderly men. (**C**) Mean hand grip and (**D**) gait speed in young, middle-aged and elderly women. ** P ≤0.01 vs 18-39y, *** P <0.001 vs 18-39y, ^###^ P <0.001 vs 40-59y.

The handgrip strength of men gradually decreased with age, and the pair wise comparisons between young, middle-age and elderly age groups were statistically significant (P<0.001, Figure 3A). Moreover, the gait speed significantly decreased with age among both men and women (P<0.001, Figure 3B). In females, handgrip strength gradually decreased with age, but the differences were not statistically significant between young and middle-aged women (P>0.05, Figure 3C). The handgrip strength of both young and middle-aged women was significantly higher compared to the elderly women (P<0.001, Figure 3D).

## DISCUSSION

Currently, there is no consensus regarding the diagnostic criteria for sarcopenia. Hence, sarcopenia diagnosis remains a matter of debate with the existence of multiple diagnostic criteria proposed by different medical expert groups. We analyzed 1685 individuals in three different age groups according to 5 diagnostic criteria for sarcopenia. The prevalence was 2.61%-9.72% among those aged over 60, 4.87%-16.37% among those aged over 65, and 18.52%-55.56% among those aged over 80. This demonstrates that the prevalence of sarcopenia increases significantly with age. Our data is consistent with previously reported research [4, 5, 7] and suggests an imbalance between muscle synthesis and degeneration [24, 25]. However, the wide-range of the cut-off points for different diagnostic parameters affects clinical determination of sarcopenia prevalence, especially among women. The cut-off points for the diagnostic parameters are determined based on the normative population data whenever available or from the predictive population data when normative data is not available. We observed that the muscle mass index of the young subjects in our study was lower than that of other Asian populations. These differences suggest that skeletal muscle mass depends on variables such as race, body shape, lifestyle, diet, and cultural background [26–28]. These differences may also be due to insufficient sample size in our study.

The variation in the muscle mass and strength in older individuals is related to the rate of muscle degeneration and the peak muscle mass and strength gained during the early years of life [29]. Muscle mass peaks around the age of 35 years [30]. After the age of 50 years, muscle mass and strength in the legs decrease every year by 1-2% and 1.5-5%, respectively [31]. In general, muscle mass reduces by about 30% and the cross-sectional muscle area shrinks by about 20% between 20 and 80 years of age [32].

Factors such as decreased nutrient intake, low physical activity, and chronic diseases increase the rate of sarcopenia progression [33]. We focused on the muscle mass of young individuals as a reference group to determine the cut-off points. In this study, we recruited healthy young women with a normal BMI and their skeletal muscle index was compared with that of the middle-aged and elderly women [34]. The middle-aged women in our study showed higher muscle mass than the younger women in our study, which was contradictory to previous findings that muscle mass reduces gradually with age. This may indicate that loss of muscle loss may occur during early periods of life in young women. It is also plausible that lifestyle and reduced physical activity may account for lower muscle mass in younger women compared to the middle aged women in our study. Larsson reported that the loss of the larger and faster contracting type II muscle fibers begins with adulthood [35]. The rate of muscle loss is significantly increased in individuals with immobility [36], including those who are immobilized at a young age. [37]. Evidence suggests that sarcopenia begins as early as the 4^th^ decade of life [29, 30, 38]. Therefore, our study demonstrates that it is not suitable to use young subjects as references to determine the SMI (skeletal muscle mass index) cut-off points for sarcopenia.

In addition to the reducing synthesis of muscle protein, increased muscle protein breakdown affects muscle loss in the elderly over 70 [14]. So we chose women aged 60-69 as comparison, it can be seen from Table 3 that the SMI value of women aged 60-69 is higher than that of women aged 18-29 and others (18-39, 30-39, 40-49, 50-59), so 18-59 years old are not significant different statistically. In this study, the women aged 60-69 are more suitable than younger women as the reference population for the muscle mass of elderly women with sarcopenia.

This study has several limitations. Firstly, because of the limited number of samples, we fail to calculate sarcopenia prevalence based on more specfic stratification on gender-age. Secondly, this study was conducted in the coastal cities of the Zhejiang Province in China. Hence, the conclusions of this study may not apply to elderly populations in other regions of China and the world. Finally, there may be significant differences in nutrition and physical fitness of individuals belonging to different age groups, which may have influenced the results of our study. Moreover, generation differences may cause discrepancies among young and elderly individuals that have been selected by random sampling of the same population. Therefore, the cut-off point represented by two standard deviations below the average muscle mass of young adults may not be accurate.

In the future, the cut-off points for the diagnosis of sarcopenia need to be verified in the Chinese population, especially those regarding physical fitness and SMI values. Therefore, we recommend large-scale, multi-center studies in the rural, urban, and inland populations. Another plausible approach to resolve the discrepancies in the SMI values for diagnosing or evaluating sarcopenia in experimental studies is to determine a suitable reference range using the SMI values of healthy elderly individuals (For example, the cut-off points for the abnormal SMI of women is one standard deviations below the mean SMI values in females aged 60-69 years). Furthermore, BMI may not fully reflect the comparable physical status of elderly individuals when compared with middle-aged and younger individuals because of decreased height, reduced muscle mass, and increased fat content. Therefore, we recommend analyzing body composition annually for the elderly and compare changes in their muscle mass over time. Such an analysis can alert patients that are susceptible to sarcopenia when the rate of muscle loss exceeds the threshold range and preventive measures can be taken. We also suggest collecting body composition analysis data of the young and middle-aged individuals in addition to the elderly in order to develop large-scale data to support diagnosis of sarcopenia. In summary, our study shows that the prevalence of sarcopenia increases gradually with age. We also demonstrate that sarcopenia prevalence varies significantly between the five diagnostic criteria used in this study. Moreover, the mean SMI values of young women was lower than the middle-aged women’in this study and was not suitable as a reference population in determining cut-off values for the diagnostic parameters in evaluating sarcopenia.

## MATERIALS AND METHODS

### Inclusion and exclusion criteria of study subjects

This observational study included 1685 volunteers aged 18 and above from two communities in the coastal cities of Zhejiang Province, China between September 2018 and June 2019. This included 422 volunteers over 60 from the elderly health monitoring cohort, which has enrolled 20,000 people from 11 cities in the Zhejiang Province and has been followed-up since 2014. 1263 healthy volunteers between 18-59 years were recruited from the same communities. We obtained written consent from all the study subjects. This study was approved by the Ethics Committee of Zhejiang Provincial Center for Disease Control and Prevention. We excluded individuals with cognitive impairment, mental illness, communication inability and cardiac pacemakers.

### Measurement of skeletal muscle mass

Skeletal muscle mass was measured using bioelectrical impedance analysis (BIA) with the InBody S10 body composition analyzer (InBody Co., Ltd, Seoul, South Korea). During testing, the study subjects adopted an erect position on an empty stomach.

### Measurement of handgrip strength (HS)

Muscle strength was measured by estimating the handgrip strength of the main hand twice with a hand-held dynamometer instrument. The higher value was used for analysis.

### Measurement of physical performance

Physical performance was assessed by estimating the gait speed (GS). The participants walked along a straight path for more than 8 meters at their usual speed and the gait speed was calculated for the middle 6-meter course.

### Diagnosis of sarcopenia

Sarcopenia was diagnosed based on the estimation of five different criteria, namely, local Standard, EWGSOP1 [1], EWGSOP2 [16], AWGS 2014 [11] and AWGS 2019 [17] as shown in Table 4.

**Table 4:** Criteria for the diagnosis of sarcopenia.

**Diagnosis is based on documentation of criterion 1 plus (criterion 2 or criterion 3)**					
	local standard*	**EWGSOP 1**	EWGSOP 2**	**AWGS(2014)**	**AWGS(2019)**
1.Muscle mass(BIA)	male <6.4 kg/m2 female <5.0 kg/m2	male <6.4 kg/m2 female <5.0 kg/m2	male <6.4 kg/m2 female <5.0 kg/m2	male <7.0 kg/m2 female <5.7 kg/m2	male <7.0 kg/m2, female <5.7 kg/m2
2.Muscle strength	male <26 kg, female <18 kg	Male <30kg, female <20kg	male <27 kg, female <16 kg	male <26 kg, female <18 kg	male <28 kg, female <18 kg
3. Performance	GS≤0.8m/s	GS ≤0.8m/s	——	GS <0.8m/s	GS <1.0m/s

local Standard*: In reference to EWGSOP1, the SMI of the local Standard is 2 standard deviations below the mean of young adults aged 18-39 in the study groups (male <6.4 kg/m2 female <5.0 kg/m2) and the gait speed at ≤0.8m/s. The handgrip strength was modified compared to AWGS 2014 and was below 26 Kg for males and below 18 Kg for females. EWGSOP2**: The SMI of the local Standard was 2 standard deviations below the mean of young adults aged 18-39 in the study group (male<6.4 kg/m2 female<5.0 kg/m2). The handgrip strength was below 27 Kg for males and <16 Kg for females.

### Data collection

We estimated the muscle mass, strength and function in 1685 volunteers by measuring body composition, handgrip strength and gait speed, respectively. The study subjects were divided into 3 age groups, namely, 541 individuals aged between 18-39 years (Average age: 28.49 ± 6.402), 722 individuals aged between 40-59 years (Average age: 49.99 ± 5.147), and 422 individuals aged 60 years and above (Average age: 67.84 ± 6.861).

### Statistical analyses

Fisher’s exact test was used to compare differences in sarcopenia prevalence between different age groups. Pearson correlation analysis was performed to determine the relationship between various indicators of sarcopenia and age. Single Factor ANOVA was used to compare the differences in skeletal mass index (SMI) between the age groups, and Tukey’s multiple comparisons test was applied for pairwise comparison. Statistical analysis was performed using SPSS software version 20.0 (SPSS Inc., Chicago, Illinois, USA).
